# Metabarcoding of Soil Fungal Communities Associated with Alpine Field-Grown Saffron (*Crocus sativus* L.) Inoculated with AM Fungi

**DOI:** 10.3390/jof7010045

**Published:** 2021-01-12

**Authors:** Íris Marisa Maxaieie Victorino, Samuele Voyron, Matteo Caser, Alberto Orgiazzi, Sonia Demasi, Andrea Berruti, Valentina Scariot, Valeria Bianciotto, Erica Lumini

**Affiliations:** 1 Institute for Sustainable Plant Protection, National Research Council (CNR), Viale Mattioli 25, 10125 Torino, Italy; irisvictorino@gmail.com (Í.M.M.V.); samuele.voyron@unito.it (S.V.); andrea.berruti@gmail.com (A.B.); valentina.scariot@unito.it (V.S.); valeria.bianciotto@ipsp.cnr.it (V.B.); 2 Department of Life Sciences and Systems Biology, University of Turin, Viale Mattioli 25, 10125 Torino, Italy; 3 Biological Science Department, Science Faculty, Eduardo Mondlane University (UEM), Av. Julius Nyerere nr 3453 Campus Principal, Maputo, Mozambique; 4 Department of Agricultural, Forest and Food Sciences, University of Torino, Largo Paolo Braccini 2, 10095 Grugliasco, Italy; matteo.caser@unito.it (M.C.); sonia.demasi@unito.it (S.D.); 5 European Commission, Joint Research Centre, Via E. Fermi, 2749, 21027 Ispra, VA, Italy; alberto.orgiazzi@ec.europa.eu

**Keywords:** soil fungal metabarcoding, saffron cultivation, arbuscular mycorrhizal fungi, AMF inocula, alpine field environments

## Abstract

Soil fungi strongly influence ecosystem structure and functioning, playing a key role in many ecological services as decomposers, plant mutualists and pathogens. Arbuscular mycorrhizal fungi (AMF) establish mutualistic symbiotic associations with plant roots and act as biofertilizers by enhancing plant nutrients and water uptake. Information about the AMF association with *Crocus sativus* L. (saffron) and their impact on crop performances and spice quality has been increasing in recent years. Instead, there is still little data on the biodiversity of soil microbial communities associated with this crop in the Alpine environments. The aims of this study were to investigate the fungal communities of two Alpine experimental sites cultivated with saffron, and to rank the relative impact of two AMF inocula, applied to soil as single species (R = *Rhizophagus intraradices*, C. Walker & A. Schüßler) or a mixture of two species (M = *R*. *intraradices* and *Funneliformis mosseae*, C. Walker & A. Schüßler), on the resident fungal communities which might be influenced in their diversity and composition. We used Illumina MiSeq metabarcoding on nuclear ribosomal ITS2 region to characterize the fungal communities associated to *Crocus sativus* cultivation in two fields, located in the municipalities of Saint Christophe (SC) and Morgex (MG), (Aosta Valley, Italy), treated or not with AMF inocula and sampled for two consecutive years (Y1; Y2). Data analyses consistently indicated that Basidiomycota were particularly abundant in both sites and sampling years (Y1 and Y2). Significant differences in the distribution of fungal taxa assemblages at phylum and class levels between the two sites were also found. The main compositional differences consisted in significant abundance changes of OTUs belonging to Dothideomycetes and Leotiomycetes (Ascomycota), Agaricomycetes and Tremellomycetes (Basidiomycota), Mortierellomycetes and Mucoromycetes. Further differences concerned OTUs, of other classes, significantly represented only in the first or second year of sampling. Concerning Glomeromycota, the most represented genus was *Claroideoglomus* always detected in both sites and years. Other AMF genera such as *Funneliformis, Septoglomus* and *Microdominikia,* were retrieved only in MG site. Results highlighted that neither sites nor inoculation significantly impacted Alpine saffron-field fungal communities; instead, the year of sampling had the most appreciable influence on the resident communities.

## 1. Introduction

Soil has always been known to be a source of microorganisms and although studied for many years its microorganism diversity is far from being fully known. Microbial communities present in soil are responsible for carrying out key ecosystem services for life on our planet but unfortunately, many beneficial functions are threatened due to climate change, soil degradation and agricultural exploitation [1,2]. The availability of soil nutrients to plants is key to obtaining healthier and better fitted crops, as they are agricultural productivity dependent on a wide range of ecosystem services provided by the soil microbiota [3]. However, most crops of global and local interest are still heavily dependent on the use of fertilizers and other chemicals which are hazardous to human and animal health and to the soil itself [4,5]. As concerns have been raised about the impact on soil microbiome of herbicides, pesticides and inorganic fertilizers, and more generally on the soil nutrient availability and plant phytotoxicity, the interest in alternative strategies for ecosystem management has greatly increased [6]. Plant growth promoting microorganisms (PGPM), i.e., soil and rhizosphere-inhabiting microorganisms, in minute quantities, promote plant growth [7], and have become one of the most important components of bio-fertilizers for sustainable agriculture. They are applied due to their role in plant growth promotion by regulating the dynamics of various processes (e.g., decomposition of the organic matter), the accessibility of various nutrients to plants (iron, magnesium, nitrogen, potassium and phosphorus), as well as acting against pathogens [8,9].

Within the categories of PGPM are the plant growth-promoting rhizobacteria (PGPR) and among the beneficial fungi, the arbuscular mycorrhizal fungi (AMF) [10,11]. It is well known that fungi can interact with plant roots in different ways, from mutualistic mycorrhizal symbioses (i.e., when both organisms live in direct contact with each other and establish mutually beneficial relationships [12], to parasitism [13]). Among mycorrhizal symbioses, arbuscular mycorrhizal (AM) fungi represent a fungal mutualistic endophytic group which establishes symbioses with over 90% of all plant species since the origin of terrestrial plants [12]. There is an increasing interest for the use of AM fungi to promote sustainable agriculture, considering the widely accepted benefits of the association with plants to nutrition efficiency (for both macronutrients, especially phosphorus, and micronutrients), water balance, and biotic and abiotic stress protection [14]. Successful progresses in AMF inoculation have been achieved and reported worldwide, but studies regarding its application from laboratory and greenhouse to field trials are still encouraged.

Agricultural practices strongly affect soil physical and chemical properties, and impact on the microbial communities affecting their abundance, diversity, and activity [15]. The effects of management practices could be positive or negative [16,17]. On the negative side, they may affect the interaction between different microbial communities, including bacteria and fungi [18], which are known to be key drivers for a more sustainable soil management [19].

High-throughput DNA sequencing techniques have greatly expanded our capability to characterize soil microbiome and identify the factors, including land management, that shape soil microbial communities across space and time [20]. In light of these studies, although most soil microorganisms still remain undescribed, some of them have recently been characterized based on their ecological strategies [21]. This aspect is of importance to identify and predict functional attributes of individual taxa that could be manipulated and managed to maintain or increase soil fertility and crop production under severe threats, including intensive exploitation [22].

While studying a soil sample it is important to consider that there is no “typical” soil microbiome, but the relative abundances of major prokaryotic and eukaryotic taxa found in the soil microbiome can vary considerably depending on the soil in question [23]. It has been widely reported that soil samples, collected from the same sampling sites just a few centimeters apart from each other, may retain very different microbiomes [20,24]. The microbiome variation can be attributed to spatial variability in the soil environment and to specific characteristics of the sampling site, sampling time and crop species and management [3]. For this reason, over the last years, some protocols have been endorsed by international projects, such as the Earth Microbiome Project (http://earthmicrobiome.org/), to analyze and compare soil microbial diversity at a large scale [25].

Fungi are widely distributed among all terrestrial ecosystems with a huge biodiversity and ecological importance by their principal role in ecosystems processes such as carbon cycling, plant nutrition, and phytopathology [26]. However, the distribution of fungal species, phyla, and functional groups as well as the determinants of fungal diversity and biogeographic patterns are still poorly understood despite recent large-scale sampling campaigns [27]. So far, information on soil fungal biodiversity in ecosystems such as Italian Alpine cultivated areas is still scarce, with the exception of some studies on vineyards [28,29] and apple orchards [30], compared to those concerning European alpine meadows, pastures, woods or specific alpine endemic plants and environments [31,32,33,34]. In the Aosta Valley region (north west Italy), smallholder farming systems, such as saffron cultivation, lead to interesting and unique Alpine agricultural ecosystems. Indeed, they are characterized by a high level of agricultural diversity, being mainly focused on meeting farmers’ needs. In this regard, they could represent a valid means for increasing incomes of multifunctional farms, with a positive impact on the recovery and economy of these often remote areas [35,36]. In particular, saffron is gaining increasing attention as an alternative crop in sustainable agricultural systems due to its unique biological, physiological, and agronomic traits, such as the capability to exploit marginal land. The application of AMF or PGPR inocula has also found interest due to the possibility to increase the overall cultivation sustainability and quality [37,38,39] In this context, we have already gained insight into the impact of AMF inoculants on growth and secondary metabolites production in saffron plantations [40].

Specifically, we record an increasing interest in applying low-input cropping systems (e.g., saffron cultivation) in mountain regions. Nonetheless, this is accompanied by a scant data collection on soil microbial diversity in saffron productive areas along with limited information on the effect of AMF inoculation on field-grown *Crocus sativus* L. (saffron). To fill this knowledge gap, the aims of this study were to investigate the fungal communities of two Alpine experimental sites cultivated with saffron, and to rank the relative impact of two AMF inocula, applied to soil as single species (R = *Rhizophagus intraradices*, C. Walker & A. Schüßler) or a mixture of two species (M = *R. intraradices* and *Funneliformis mosseae*, C. Walker & A. Schüßler), on the resident fungal communities which might be influenced in their diversity and composition. We used Illumina MiSeq metabarcoding on nuclear ribosomal ITS2 region to characterize the fungal communities associated to Crocus sativus cultivation in two fields, located in the municipalities of Saint Christophe (SC) and Morgex (MG), (Aosta Valley, Italy), treated or not with AMF inocula and sampled for two consecutive years (Y1; Y2). In the frame of an increasing demand to reduce chemical inputs in agriculture, the results of this study could reveal useful information on the real impact of AMF inoculation on the resident fungal communities opening new perspectives on the possible roles of AMF and/or other most competitive beneficial microbes to be further exploited in a sustainable agriculture perspective.

## 2. Materials and Methods

### 2.1. Sampling Sites

**Figure 1:**
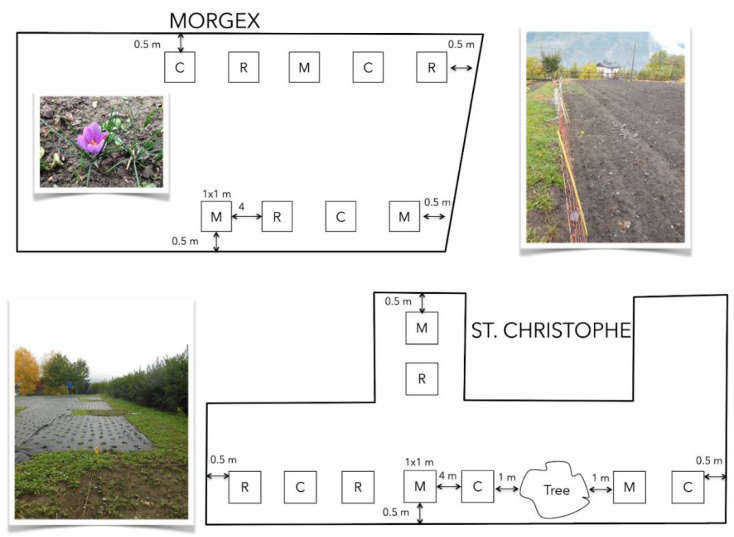
Scheme of the randomized block design in the experimental sites located in the municipality of Morgex (45°45′35.1″ N; 7°02′37.3″ E, 1000 m a.s.l.) and Saint Christophe (45°45′06.9″ N; 7°02′37.0″ E, 700 m a.s.l.), Aosta Valley (AO), Italy. (C = Carrier (Control); R = Single-species inoculum (*Rhizophagus intraradices*); M = Multi-species inoculum *(R. intraradices* and *Funneliformis mosseae*).

Two Italian western alpine experimental sites, located in the municipality of Morgex (45°45′35.1″ N; 7°02′37.3″ E; 1000 m a.s.l.) and Saint Christophe (45°45′06.9″ N; 7°20′37.0″ E; 700 m a.s.l.) (Aosta Valley, AO) and cultivated with saffron for at least the previous three years, were selected for our analyses (Figure 1). The experiment covered two successive cultivation cycles: year 1 (Y1) (2016–2017) and year 2 (Y2) (2017–2018) as reported by Caser et al. [40]. Before starting the experiment, both fields were milled and, at time of plantation (mid-August), *Crocus sativus* corms (caliber size of 6–7 cm, without antifungal application), kindly provided by the Azienda Agricola “La Branche di Diego Bovard” (Morgex, AO, Italy), were treated with two AMF inocula (MycAgro Lab, Breteniére, France). One was composed by the single species *Rhizophagus intraradices* (R), the other by the mix of *R. intraradices* and *Funneliformis mosseae* (M). As Control (C), corms were not inoculated with AMF but with their sterile carrier. Just before the first year of planting (August 2016), 10 g of inoculum/carrier was placed under the corms to ensure contact between plant and the treatment [40], while in the second year (August 2017) corms were planted without any further inoculum supply.

A randomized block design was used, with three experimental plot units (blocks) (Figure 1). Each plot unit consisted of 56 corms, planted in a 1.44 m^2^ area (39 corms m^2^). Inter-row planting distance was of 7 cm, while between-row distance was of 25 cm. Plots were separated from each other with at least 4 m distance. Irrigation was provided when needed and hand weeding control was conducted during cultivation, while no pre-planting fertilization, tillage, or treatments against pathogens were applied. Sites were characterized by semi-continental climate, with a long and cold winter, and both of them had a sandy-loam texture, according to the USDA classification, and similar chemical characteristics [41]. At each experimental site, samples were collected for 2 following cultivation cycles (Y1 and Y2) at flowering time (November 2016 and 2017). Five replicates of bulk soil were collected in each of the three experimental plot unit (C; M; R) with an earth drill from the first 30 cm of soil following a V-shaped sampling pattern, pooled to generate a total of three biological replicates and kept in zip lock bags at 4 °C before being processed in the laboratory. Soil samples were sieved using 2 mm stainless steel sieve mesh and then put in tubes at −20 °C for further molecular analysis. A total of 9 soil samples for Y1 and 9 samples for Y2 were collected respectively in Morgex and Saint Christophe for a grand total of 36 soil samples.

### 2.2. Site Environmental Conditions

In the first cultivation season (2016–2017), average temperatures ranged from −3.5 °C to 23.4 °C in Saint Christophe, and from −2.7 °C to 20.0 °C in Morgex. In particular, Morgex showed greater precipitation rate (57.5 mm/month) and higher relative humidity (R.H.), with the peak in November (89.3%) compared to Saint Cristophe. Conversely, the total radiation was generally higher in Saint Cristophe, from −2.3 °C to 22.6 °C than in Morgex, from −1.2 °C to 19.5 °C. In fact, Morgex showed, in general, more wet conditions and more rainfall weather conditions, with the highest precipitation rate and R.H. (74.5 mm/month and 84.0%, respectively).

### 2.3. Soil DNA Extraction, PCR Amplification and Sequencing

In order to achieve our main objective, DNeasy PowerSoil Kit, (formerly sold by MO BIO as PowerSoil DNA Isolation Kit) (Qiagen, Hilden, Germany) endorsed by the Earth Microbiome Project (http://earthmicrobiome.org/) was used. Extractions were carried out from 250 mg of soil samples, following the manufacturer’s protocol.

To investigate the total fungal community, the nuclear ribosomal ITS2 region was amplified using Invitrogen Platinum HotStart PCR Master MIx (Thermo Fisher Scientific, Waltham, Massachusetts, USA) from all DNA extracts by means of a semi-nested PCR approach. In the first PCR, the entire ITS (ITS1-5.8S-ITS2) region was amplified with the generic fungal primer pair ITS1F-ITS4 [42,43]. The cycling conditions were an initial step at 95 °C for 15 min, 35 cycles at 95 °C for 35 s, 57 °C for 35 s, 72 °C for 45 s, and a final extension step of 72 °C for 7 min. Each PCR product was checked on agarose gel, diluted at 1:20 and used as a template in the semi-nested PCR targeting the ITS2 region, with primer fITS9 (5′– GAACGCAGCRAAIIGYGA–3′), which has been previously reported to match only 20% of 5.8S AMF sequences at NCBI [44], and ITS4 (5′–TCCTCCGCTTATTGATATGC–3′). This second couple of primers was added to Illumina overhang adapter sequences: forward overhang: 5′-TCGTCGGCAGCGTCAGATGTGTATAAGAGACAG- [locus specific target primer], reverse overhang: 5′ GTCTCGTGGGCTCGGAGATGTGTATAAGAGACAG- [locus specific target primer]. The semi-nested PCR cycling conditions were an initial step at 95 °C for 15 min, 27 cycles at 95 °C for 30 s, 57 °C for 30 s, 72 °C for 30 s, and a final extension step of 72 °C for 7 min.

For the 36 samples (18 in Y1 and 18 in Y2) DNA extracted was amplified in triplicate and pooled prior the purification using the Wizard^®^ SV Gel and PCR Clean-Up System (Promega, Milano, Italy). A final number of 36 PCR purified products were quantified with Qubit 2.0 (Invitrogen, Carlsbad, California, USA) following manufacturer’s protocol and sent for Illumina MiSeq sequencing (2 × 250 bp) to IGA technologies (Udine, Italy).

### 2.4. Bioinformatic and Statistical Analyses on Soil Fungal Communities

DNA reads from each sampling point included forward and reverse sequences in separate files. Sequencing adapters and primers were removed, and the sequences were then analyzed by means of the microbiome bioinformatics platform QIIME2 (Quantitative Insights Into Microbial Ecology 2, version 2019.7 [45]. Denoising and quality control, including removal of chimeras, were achieved by means of the DADA2 [46] plugin (qiime dada2 denoise-paired). To avoid low quality sequences, reads were truncated (300 bp for forward, 290 bp for reverse reads). The classifier adopted for the taxonomic assignment of the total fungal community was generated using the UNITE Community (2019): UNITE QIIME release for Fungi version 02.02.2019.

The generated dataset, including OTU table, taxonomy table and metadata, was then imported in Rstudio (RStudio Team 2016) and was used to create a phyloseq object with the R package phyloseq [47] that was employed for all the following analyses. To allow statistical comparisons, the OTU table was rarefied at 8185 sequences per sample, by means of the rarefy_even_depth function of the R package phyloseq. Rarefaction curves of the non-rarefied and rarefied OTU table were obtained by means of the function rarecurve of the R package vegan v. 2.5-4 [48]. Biodiversity analyses were carried out by comparing the richness (number of species) and evenness (richness taking into account relative abundances) of fungal communities. Within-sample (alpha) diversity was assessed by three estimators: “observed fungal species,” “Chao1,” and “Shannon”. The alpha diversity indices were calculated and plotted by means of the functions estimate_richness and plot_richness implemented in the R package phyloseq [47]. Bar plots were then generated with the R package ggplot2 version 3.1.0 [49]. Ordination plots (NMDS) were generated by means of the R packages phyloseq, ggplot2 and plyr version 1.8.4 [50]. The trophism of retrieved OTUs was defined by means of the FUNGuild package [51]. In order to evaluate significant differences (*p* < 0.05) between alpha diversity indexes or influences of the site, inoculum and time of sampling over the fungal populations’ distributions, the adonis function from R package vegan was used to perform PERMANOVA tests. PERMANOVA tests were run for every metadata, or combinations of metadata using dissimilarity index of Bray-Curtis. Permutest was also run in order to check the validity of the previous tests by means of the function betadisper of the R package vegan. Significant differences between taxonomic distribution at the phylum level of fungal communities retrieved from the two sites was evaluated by means of the software Past 4 (https://past.en.lo4d.com/windows), and the test Anova, Mann-Whitney pairwise comparison; Bonferroni corrected (*p* < 0.05). ITS2 representative sequences were deposited in GenBank under the accession numbers (MW162630-164623).

## 3. Results

### 3.1. Composition and Structure of Fungal Soil Communities

After the bioinformatic analysis, 487,815 high-quality ITS2 sequences (out of a total of 2,572,327 raw sequences) were retained and clustered in 1391 operational taxonomic units (OTUs). In order to perform statistical analysis, the resulting OTU tables were then rarefied at 8185 sequences per sample for a total number of 1100 OTUs. The fungal taxonomic diversity retrieved in the two sites (MG and SC) for the two years of sampling (Y1 and Y2) is reported at phylum level in Table 1.

The percentage of the different phyla was variable among the two sampled sites. As a matter of fact (Table 1), Basidiomycota were particularly abundant in the two sites sampled and for both years (Y1 and Y2). More in detail, Basidiomycota, over the two sampling times, are significantly more present in SC (approx. 59–66%; Y1 and Y2 respectively) than in MG (approx. 45–46%; Y1 and Y2 respectively). Conversely, Ascomycota were significantly more present in MG (approx. 31–28%), both for Y1 and Y2, than in SC (approx. 18–16%). A similar trend is shown by Mortierellomycota and only for Y2 by Mucoromycota. Our data showed a significant decrease of Chytridiomycota in SC at Y2, while for Kickxellomycota, Glomeromycota, and for the unidentified fungi, we did not report any significant differences.

In particular, in MG, the Basidiomycota most represented classes were Agaricomycetes (25–31%) followed by Tremellomycetes (16–13%), Pucciniomycetes (2–0.67%) and Microbotriomycetes (1.4–0.90%), in Y1 and Y2 respectively, while in SC Agaricomycetes represented up to 49–54% of the total followed by a smaller percentage of Tremellomycetes (6.6–8.5%) and a percentage less than 1–2% of both Pucciniomycetes and Microbotriomycetes. Considering Ascomycota, the most represented classes were for MG Dothideomycetes (12–7.8%) followed by Sordariomycetes (8–9%) and Leotiomycetes (5.5–7%) for Y1 and Y2 respectively. Concerning SC, the most represented class is Sordariomyctes (6–7%) followed by Dothideomycetes (6–3%) and Leotiomycetes (3.3–3%) for Y1 and Y2 respectively. The complete taxonomic association at class level is reported in Table S1.

**Figure 2:**
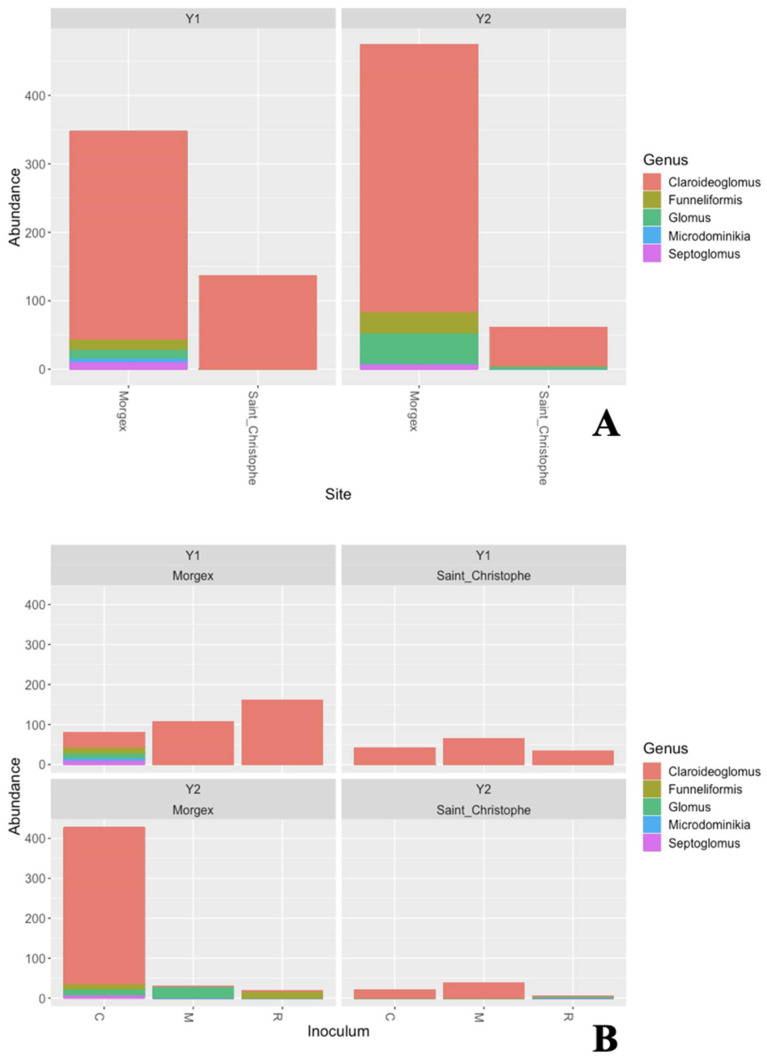
(**A**) Bar chart showing the taxonomic distribution (based on fungal ITS2), at genus level, of OTUs belonging to the phylum Glomeromycota, retrieved from Morgex, and Saint Christophe; (**B**) Bar chart showing the effect of the inoculum on the taxonomic distribution (based on fungal ITS2), at genus level, of OTUs belonging to the phylum Glomeromycota, retrieved from Morgex, and Saint Christophe. (Y1: year 1; Y2: year 2; C = Carrier (Control); R = Single-species inoculum (*Rhizophagus intraradices*); M = Multi-species inoculum (*R. intraradices* and *Funneliformis mosseae*).

Regarding Glomeromycota, our results underline that, when considering site and time of sampling, there were not significant differences in terms of taxa abundance while AM fungal community composition was variable in terms of retrieved genera (Figure 2A,B). As a matter of fact, SC was mostly dominated by the genus *Claroideoglomus* both for Y1 and Y2, whereas MG showed higher diversity: *Claroideoglomus* was again the most represented genus, but in MG we retrieved also *Funneliformis, Septoglomus, Glomus* and *Microdominikia* genera.

Community shifts from Y1 and Y2, due to inoculation with AMF, are shown in Figure 2B. In SC all the treatments displayed lower relative abundance levels of the most represented genus (i.e., *Claroideoglomus*) which was nearly to disappear only in the single species inoculated plots (R). In MG, the abundance of *Claroidoglomus* OTUs sharply decreased in both the inoculated plots (M and R) giving way to OTUs belonging to *Glomus* and *Funneliformis* genera, which were not retrieved in the Y1. On the other hand, in the MG control plot (C), the abundance of *Funneliformis*, *Glomus* and *Septoglomus* remained almost unchanged, *Microdominikia* instead was no longer detected and *Claroidoglomus* represented the most retrieved genus ever found.

**Figure 3:**
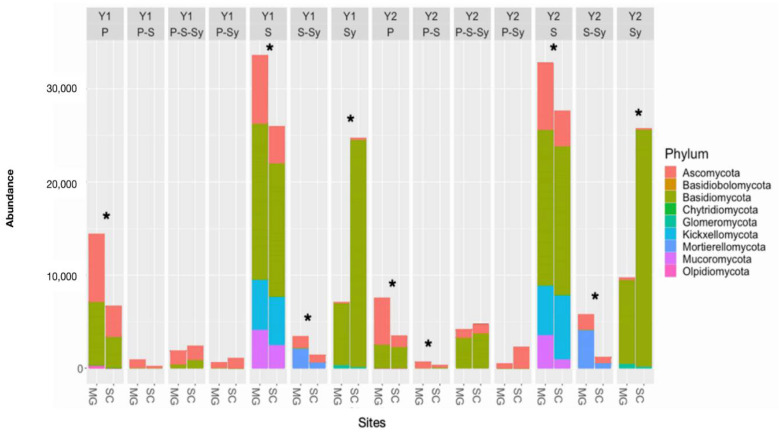
Trophic distribution and phylum composition of OTUs retrieved from Morgex (MG) and Saint Christophe (SC) at sampling time Y1 (first year) and Y2 (second year). S, saprotrophs; Sy symbiotrophs; P, pathotrophs (fungi showing different trophic behaviors are separately annotated). Data are expressed as percentage (mean ± standard deviation). *: indicate significant differences (Anova; Mann-Whitney pairwise comparison; Bonferroni corrected *p* values; *p* < 0.05).

The ITS region sequence data were also used to infer the putative ecological roles of the total fungal communities at each sampled site (Figure 3). The assignment to a strategy was possible for 546 (49, 63%) out of 1100 ITS2 OTUs. In terms of trophic diversity, the abundance of saprotroph, pathotroph and saprotroph/symbiotroph fungi was significantly higher in MG than in SC, both at Y1 and Y2, and only for pathotroph/saprotroph guilds at Y2. Symbiotrophs are instead significantly more represented at SC, both at Y1 and Y2.

### 3.2. Soil Fungal Microbiota Assembly and AMF Inocula Impact

**Figure 4:**
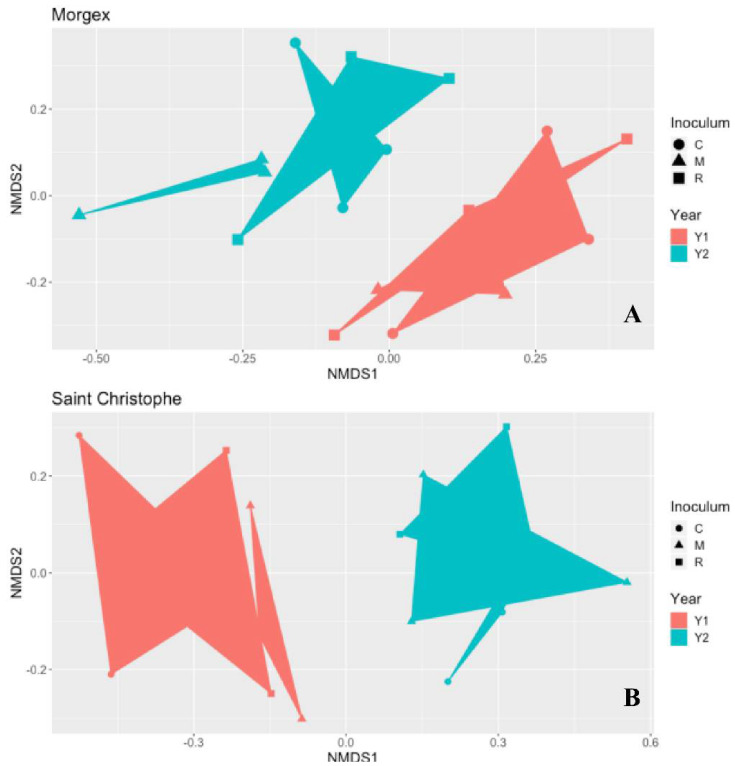
Ordination plots (NMDS) showing beta diversity of fungal communities of Morgex (**A**) and Saint Christophe (**B**). The two fungal communities are significantly affected by the sampling time (Y1; Y2) (*p* ≤ 0.05, Permanova; Bray–Curtis) but not by the inoculum (C = Carrier Control; R = Single-species inoculum (Rhizophagus intraradices); M = Multi-species inoculum (R. intraradices and Funneliformis mosseae).

The evaluation of alpha diversity highlighted that the variable most significantly affecting the fungal community was the year of sampling (Y1 and Y2), while AM fungal inocula did not exert significant effect (Figure S1). The homogeneity of dispersion among groups was supported by a non-significant result in permutest. PERMANOVA analyses also revealed that the fungal communities thriving in the soil of Morgex and Saint Christophe were not significantly affected by the AMF inocula but only by the sampling time (Y1; Y2) (*p* ≤ 0.05, Bray–Curtis) (Figure 4).

## 4. Discussion

Despite growing interest, the variability of soil microbial fungal communities (fungi and bacteria) and the biotic and abiotic factors that drive their differentiation are still poorly understood in some remote, but still ecologically important, environments such as Alpine marginal cultivated fields. Soil microbiome could be of particular relevance in saffron (*Crocus sativus* L.) cultivation, since soil has been shown to serve as a reservoir of microorganisms which, once colonizing roots, might contribute to saffron plant growth, nutrient availability and pathogen defense [38,39,52].

Besides bacteria, the major component of soil microbiota is represented by fungi which play crucial roles as saprotrophs, plant mutualists, symbionts and pathogens [53]. Moreover, fungi are key in controlling the soil structure and its water content and in regulating the aboveground biodiversity and productivity [54]. Due to their large number of species, specialization, and important ecological functions, fungi are also considered excellent bioindicators of soil quality [55]. This aspect is especially relevant in the case of marginal alpine areas where saffron yield and quality may vary greatly by site on the basis of several factors such as soil types, climatic conditions and cultivation techniques [35,36]. However, one of the most important factors, namely the soil microbiota associated with cultivation sites, has been poorly explored in *C. sativus* worldwide, in spite of its economic value both at national and international level. Here, for the first time, we reported results obtained by profiling the fungal components of soils cultivated with saffron through Illumina MiSeq metabarcoding on fungal ITS2 from the Valle d’Aosta agriculture sites of Saint Christophe (SC) and Morgex (MG), and covering two years of sampling (Y1, Y2).

In general, the two sites were characterized by slightly different fungal phyla assemblages: in both sites and years (Y1 and Y2), Basidiomycota were particularly abundant in soil. This findings is not in line with previous reports identifying dominant fungal phylotypes as belonging to generalist Ascomycota that dominate soils globally [56]. Our results are also different from those obtained by Coller et al. [29] in Alpine vineyards soils showing Ascomycota (51.8%) and Zygomycota (20.1%) as dominant Phyla, while Basidiomycota representing only a small fraction (11.2%). The surprising percentage of Basidiomycota in saffron cultivated fields, where some of the OTUs were affiliated to ectomycorrhizal species (e.g., Inocybe vulpinella Bruylants; Suillus granulatus (L.) Roussel, S. viscidus (L.) Roussel), could be explained by the fact that most major genera of fungi, such as the ectomycorrhizal genera *Russula*, *Boletus*, *Inocybe*, *Cortinarius* and *Amanita*, seem to be present on all habitable continents [56]. It is worth noting that the site of SC is also characterized by: a shrubby fence, the presence of a tree inside the plot, several plants of birch just around the plot fence and very few grass (Figure 1). We can speculate that our data on the belowground fungal community may provide useful elements on the aboveground features such as previous and actual vegetation coverage and/or agronomic procedures, allowing to assess the impact of anthropogenic land use to hidden diversity in soil [31]. Through the analysis of fungal ecological guilds, we indeed highlighted that fungal symbiotrophs were significant more in SC than in MG. On the other hand, the higher abundance of saprotroph, pathotroph and saprotroph/symbiotroph fungi in MG could be explained by some other site specific edaphic characteristics.

Our results are in line with those obtained in the same experimental plots by Caser and colleagues [41]. Indeed, they clearly demonstrated that the largest difference in physiological and biochemicals flower-related traits and corm properties of saffron plants cultivated in the same sites (i.e., Morgex and Saint Christophe) were between the growing seasons (Y1 to Y2). In particular, they found that many more moldy corms (wilted) occurred in the first cultivation season (36.8%) than in the second (16.8%), and more in Morgex (52.4%) than Saint Christophe (39.1%). They argued that elevated percentage of wilted corms was probably due to the high relative humidity and precipitation rate (more than 550 mm/year), mainly occurring in MG than in SC and, to the absence of corm antifungal treatments. In accordance with those findings, our results showed that Morgex’s soil seems to thrive not only significantly higher saprotroph but more important, pathotroph and saprotroph/symbiotroph fungi.

Even if a specific characterization of plant pathogens was not conducted, the high wilting rate found in MG could be related and favored by the presence, in this site, of several fungal species belonging to *Ascomycetes* such as *Blumeria*, *Colletotrichum*, *Curvularia*, *Gibberella*, *Leptosphaeria*, *Plectosphaerella*, *Ramularia*, *Stigmina*. Indeed, all these have been reported to be associated with saffron diseases [57,58]. We also detected some taxa, previously reported as endophytic fungal isolates, belonging to two Ascomycota lineages representing two orders (Helotiales and Pleosporales) and one order each in Basidiomycota and Mortierellomycota, namely Agaricales and Mortierellales, respectively. The presence in soil of microbial endophytes is very important. In fact, some endophytes (e.g., *M. alpina*) showed positive effects on many growth parameters (i.e., total biomass, size of corms, number of apical sprouting buds, number of adventitious roots) and plant secondary metabolite production [59]. Furthermore, the endophyte can enhance biotic stress tolerance to corm rot fungus by releasing arachidonic acid [60].

Despite being also designed to rank the relative impact of two AMF inocula on the resident soil fungal communities, this study highlighted that the variable significantly affecting the fungal communities was instead only the year of sampling (Y1 and Y2). Neither the sites nor inoculum application significantly influenced soil fungal diversity and composition. In particular, our results also showed that apparently, the resident AM fungal communities found in the treated or control plots of Morgex and Saint Christophe were not significantly affected by the introduction of commercial AMF-based inoculum and that the AMF sequences retrieved from the soil metagenome very partially reflected the species inoculum composition. In addition, unlike what has been previously reported [61] the inoculation process seems to increase the dominance of a single species (i.e., *Claroidoglomus*) and decrease diversity of the preexisting AMF communities. Unlike the results obtained in other Alpine environments of Northern Italy by Berruti et al. [28], on vineyards of Aosta Valley, and Turrini et al. [30], on apple orchards of South Tyrol, the most abundant genus retrieved in saffron alpine agriculture sites, was not *Glomus* but *Claroideoglomus*, representing up to 80% of the *Glomeromycota* sequences. Similar to previous reports, AMF taxa belonging to *Septoglomus* and *Funneliformis* corresponded to less than 4% of total sequences in these agriculture sites. However, we must point out that the differences found may be due to the different methods, which were used in the studies cited above, to investigate AM soil fungal communities and only partially overlapping with ours. Regarding the higher AMF abundance and biodiversity found in Morgex we could speculate that this site is characterized by some patchy areas dominated by grasses; an environment more favorable to AM Fungi such as surrounding herbaceous plants, from which AMF propagules and/or healthy AMF mycelial networks could gradually have colonized saffron cultivated fields. Another hypothesis could be that in MG the vegetation-mediated legacy effects on soil microbial communities is still maintained.

Furthermore, no sequences of *Rhizophagus/Rhizoglomus* spp. that represented the main taxon of one of the applied inoculum (i.e., R) were found. Many factors can affect the success of inoculation and AMF persistence, including environmental and cultivation conditions, species compatibility, degree of spatial competition with other soil organisms, and the time of inoculation. Hence, it is important to assess the effects of AMF on crop traits both as early application and as residual persistence in the following crop cultivation seasons [61]. This aspect of particular importance is in accordance with previous results showing that AMF root colonization of *C. sativus,* treated with mixed or with single inoculum, during the two successive cultivation cycles (year Y1 and Y2), was very low in terms of both intensity of colonization (0.0–9.0%) and percentage of arbuscules (0.0–4.4%) [40].

These results are, however, in line with those reported by Ceballos et al. [62] and Berruti et al. [61] on cassava and maize plants inoculated by AMF, respectively. In fact, even if root mycorrhization was very low for both plants (which are usually very well mycorrhized in the field) they eventually produced higher yields anyway. Beside the results regarding the belowground aspect of our experiment, it is important instead to underline that in many experiments AM fungal applications exerted an important impact on the above-grown features [37,39]. In particular, the inoculation of saffron with single or multiple AM fungal isolates was demonstrated to increase either flower production and, saffron yield as well as spice antioxidant activity and the content of some important bioactive compounds (i.e., picrocrocin, crocin I, and quercitrin) [39,40].

We can assume that the AM fungal inoculum may have had a side stimulating effect on other resident soil microbial components (i.e., PGPR) that affect plant growth and physiology. Another possible explanation could be the presence of PGPR strictly associated with spores of some AM fungal species commonly used as inoculum [63].

## 5. Conclusions

For the first time, we have characterized the fungal communities of an Italian alpine agroecosystem cultivated with saffron and treated with arbuscular mycorrhizal fungal inocula. Since saffron is the world’s highest priced spice, the increases in yield and quality obtained using AMF inoculum along with having no evident impact on resident microbial populations suggests that farms in marginal areas such as alpine sites can increase profitability by inoculating saffron bulbs with arbuscular mycorrhizal fungi.

In addition to AMF, it would be advisable to investigate other endophytes to be co-inoculated with saffron bulbs, or directly recruited from soils, that could offer further advantages, for example, an increasing tolerance to biotic and/or abiotic stresses. This last aspect is of particular importance because, after a few years of continuous cultivation, saffron could exert allelopathic activity against many soil components [64,65], thus compromising both its replanting and the cultivation of alternative crops (i.e., lettuce) [66].

Lastly, the provided datasets may contribute to future searches on fungal bio-indicators to be applied as biodiversity markers of a specific site and/or agriculture cultivation.

## Figures and Tables

**Table 1:** Taxonomic distribution, at phylum level, of operational taxonomic units (OTUs) retrieved from the two sampled sites (Morgex: MG; Saint Christophe: SC) at Y1 (year 1) and Y2 (year 2). Data are expressed as percentage (mean ± standard deviation). Different letters indicate significant differences (Anova; Mann-Whitney pairwise comparison; Bonferroni corrected *p* values; *p* < 0.05).

	Y1		Y2	
Phylum	MG	SC	MG	SC
Ascomycota	31.06 ± 5.78 ^a^	18.39 ± 1.96 ^b^	28.80 ± 7.41 ^a^	16.12 ± 4.36 ^b^
Basidiomycota	45.14 ± 7.33 ^a^	59.83 ± 6.59 ^b^	46.86 ± 11.36 ^a^	66.02 ± 6.21 ^b^
Chytridiomycota	1.46 ± 0.86 ^a^	1.14 ± 0.58 ^a^	2.01 ± 1.18 ^a^	0.66 ± 0.54 ^b^
Glomeromycota	0.50 ± 0.57 ^a^	0.22 ± 0.19 ^a^	0.68 ± 1.04 ^a^	0.29 ± 0.31 ^a^
Kickxellomycota	7.42 ± 1.89 ^a^	7.08 ± 0.75 ^a^	7.35 ± 2.08 ^a^	9.35 ± 1.52 ^a^
Mortierellomycota	2.92 ± 1.30 ^a^	0.93 ± 0.63 ^b^	5.63 ± 3.41 ^a^	0.79 ± 0.65 ^b^
Mucoromycota	5.64 ± 1.38 ^a^	3.43 ± 1.40 ^a^	4.87 ± 1.78 ^a^	1.32 ± 1.08 ^b^
unidentified	5.28 ± 2.77 ^a^	8.94 ± 4.82 ^a^	3.71 ± 1.43 ^a^	5.43 ± 5.78 ^a^

## Data Availability

The sequences data presented in this study are available in GenBank under the accession numbers (MW162630-164623).

## Supplementary Materials

The following are available online at https://www.mdpi.com/2309-608X/7/1/45/s1, Table S1. Taxonomic distribution, at Class level, of OTUs retrieved from the two sampled sites (Morgex: MG; Saint Christophe: SC) at Y1 (year 1) and Y2 (year 2); Figure S1: Alpha diversity measures of saffron fields: Observed OTUs, ChaoI and Shannon.
